# Physiological responses of maca (*Lepidium meyenii* Walp.) plants to UV radiation in its high-altitude mountain ecosystem

**DOI:** 10.1038/s41598-020-59638-4

**Published:** 2020-02-14

**Authors:** Thais Huarancca Reyes, Eliana Esparza, Gaia Crestani, Fabián Limonchi, Rudi Cruz, Norma Salinas, Andrea Scartazza, Lorenzo Guglielminetti, Eric Cosio

**Affiliations:** 1 Department of Agriculture, Food and Environment, University of Pisa, Via del Borghetto 80, 56124 Pisa, Italy; 2 Sección Química, Pontificia Universidad Católica del Perú, Av. Universitaria 1801, San Miguel, Lima 32 Peru; 3 Institute of Research on Terrestrial Ecosystems, National Research Council, Via Moruzzi 1, 56124 Pisa, Italy; 4 Interdepartmental Research Center “Nutraceuticals and Food for Health”, University of Pisa, Via del Borghetto 80, 56124 Pisa, Italy

**Keywords:** Plant physiology, Abiotic

## Abstract

Ultraviolet (UV) radiation is a small fraction of the solar spectrum, which acts as a key environmental modulator of plant function affecting metabolic regulation and growth. Plant species endemic to the Andes are well adapted to the harsh features of high-altitude climate, including high UV radiation. Maca (*Lepidium meyenii* Walpers) is a member of Brassicaceae family native to the central Andes of Peru, which grows between 3500 and 4500 m of altitude, where only highland grasses and few hardy bushes can survive. Even though maca has been the focus of recent researches, mainly due to its nutraceutical properties, knowledge regarding its adaptation mechanisms to these particular natural environmental conditions is scarce. In this study, we manipulated solar UV radiation by using UV-transmitting (Control) or blocking (UV-block) filters under field conditions (4138 m above the sea level) in order to understand the impact of UV on morphological and physiological parameters of maca crops over a complete growing season. Compared to the UV-blocking filter, under control condition a significant increase of hypocotyl weight was observed during the vegetative phase together with a marked leaf turnover. Although parameters conferring photosynthetic performance were not altered by UV, carbohydrate allocation between above and underground organs was affected. Control condition did not influence the content of secondary metabolites such as glucosinolates and phenolic compounds in hypocotyls, while some differences were observed in the rosettes. These differences were mainly related to leaf turnover and the protection of new young leaves in control plants. Altogether, the data suggest that maca plants respond to strong UV radiation at high altitudes by a coordinated remobilization and relocation of metabolites between source and sink organs via a possible UV signaling pathway.

## Introduction

The Andes form the longest and one of the highest mountain ranges in the world, with a width of approximately 400 Km and over 100 peaks above 6000 m of altitude along the west coast of South America^1^. An extensive biodiversity of plant species is endemic to this region and these have been used for centuries by the native population. Interestingly, these crops are well adapted to the harsh climate of the Andes including drought, freezing temperatures, strong wind and high ultraviolet (UV) radiation^2^. UV represents a small fraction of the solar spectrum and based on its biological effects it is divided into UVC (100–280 nm), UVB (280–315 nm) and UVA (315–400 nm). However, only UVA and fraction of UVB radiation reach the Earth surface, of which UVB has the highest energy in the solar spectrum and thus has a major impact on all organisms. Moreover, levels of UVB, as well as those of UVA, vary in time and space depending on the weather, climate and geographical conditions, such as season of the year, altitude, latitude and atmospheric surface^3^.

In the past decades, there has been an increased interest in the effects of UVB radiation on plant physiology and biochemistry. It has been reported that low doses of UVB mainly induce photomorphogenic changes via the UVB RESISTANCE LOCUS 8 (UVR8) photoreceptor, while high doses can induce the production of reactive oxygen species (ROS), reduction of photosynthesis performance, activation of additional stress signaling pathways and damage to DNA, cell membranes and proteins^4–7^. In addition, the activation of UVB-specific and/or nonspecific signaling pathways depend on the fluence rate, duration, acclimation, plant species and interaction with other environmental factors^8,9^. Besides UVR8, part of the blue light photoreceptors including phototropins (PHOT) and cryptochromes (CRY) can absorb photons in the UVA region. However, while most studies refer to plant responses to blue or UVB little is known about UVA effects, commonly assumed that it is similar to the blue light^10^.

Maca (*Lepidium meyenii* Walpers) is a Brassicaceae crop native to the central Andes of Peru, which grows at elevations of 3500 to 4500 m above the sea level where only highland grasses and few hardy bushes can survive^11^. The main edible part of maca consists of a fused taproot and hypocotyl (referred to as ‘hypocotyl’), which grows and expands during the vegetative phase and reaches full enlargement 7 months after sowing in mountain ecosystem^12^. It has been demonstrated that maca hypocotyl contains several bioactive compounds including glucosinolates, macamides, macaenes, sterols, phenolics, essential oils and polysaccharides, which are related to maca medicinal properties^13,14^. Even though many studies have been focused on its phytochemical characteristics, less is known regarding the adaptation mechanisms of maca to its particular natural environmental conditions. Our recent study revealed that maca grown in growth chamber showed differential UVB responses including a coordinated source-sink carbon allocation and a systemic phenotypical plasticity^15^. These results prompted us to realistically evaluate the physiological responses of maca to UV in its natural mountain ecosystem, which is much more complex than laboratory-simulated conditions. For this purpose, maca plants were grown in the field in the Peruvian central Andes under transmitting- or blocking-solar UV radiation conditions in order to examine morphological and physiological parameters at the middle and the end of their biological cycle.

## Materials and Methods

### Experimental site and plant growth conditions

This study was conducted in the Junín plateau (Peru) between December 2016 and June 2017, a period that spans the usual maca harvest in the region. Site coordinates were 11°09′40.6″S 75°59′01.0″W and elevation was 4138 m above the sea level. During the experimental period, temperature ranged from −4.8 to +24.3 °C and relative humidity ranged from 22 to 98% (iMini Temperature and Humidity Data Logger MX-HS-S-8-L, CRYOPAK Verification Technologies, Inc., USA). Seeds of maca (*Lepidium meyenii* Walpers) were obtained from the local farmers, and sown in the field in an area of 15 m × 15 m under six plastic film covered tunnels of dimensions 3 m length × 1.5 m width × 1.5 m height.

The tunnels were set up in the field in a N-S orientation. They were covered with either UV-blocking (block 100% UV < 350 nm and 20% Visible; referred to as UV-block) or UV-transmitting (block 40% UVB, 30% UVA and 10% Visible; referred to as Control) plastic filters (G. Valota SpA, Bergamo, Italy). The transmission characteristics of these filters were measured with a Shimadzu Spectrophotometer (UV-1800) (Supporting information, Fig. S1). The front and back end and bottom sides of the tunnels (10 cm above ground) were left uncovered to allow normal ventilation, permitting no significant differences between the temperature and humidity inside and outside the tunnels. Water was supplied as needed to prevent wilting, so that drought did not influence the variables measured along the experiment. No differences were observed in the germination rate between treatments (almost all seeds were germinated 7 d after sowing).

### Radiation measurement

Solar radiation was measured at midday. Photosynthetically active radiation (PAR) averaged 2098 µmol m^−2^ s^−1^, UVA ranged from 45 to 72 W m^−2^, and UVB ranged from 2.091 to 3.803 W m^−2^ over the whole experimental period (Skye Instruments Ltd., Powys, UK). Daily erythemal UV index data downloaded from the SENAMHI web page (http://www.senamhi.gob.pe/) ranged between 11 and 19 at the experimental site (Supporting information, Fig. S2). It should be mentioned that the erythemal UV data can be used as a proxy for plant UV exposure as previously described^16^.

### Biometric analysis

Sampling was performed in the middle (4 month-old, beginning of April 2017) and at the end (7 month-old, middle of June 2017) of the cultivation period. Rosette diameter and underground organ length were measured, as well as the leaves number. Plants were separated into epigeal, hypocotyl and roots, and then weighed (fresh weight, FW). Underground tissues were previously washed with water to remove soil. The dry weight (DW) of plant organs was registered after complete drying at 60 °C (2 weeks). Six biological replicates were considered for this analysis.

An additional set of plants not used for testing biometric traits were collected and immediately placed in metal baskets in 10 L CX-100 Taylor-Wharton containers filled with liquid nitrogen. The containers were shipped to the laboratory and remained in liquid nitrogen until used. Samples were lyophilized for further biochemical analyses.

### Total soluble sugars quantification

Total soluble sugars (TSS; sucrose, glucose and fructose) from epigeal, hypocotyl and root organs were extracted and assayed as previously reported^17^. The accuracy of the method was calibrated using standards with known amounts of carbohydrates. Recovery experiments were carried out to evaluate losses during extraction. The concentrations of standards added were similar to those estimated to be present in the tissues in preliminary experiments. The recovery ranged between 97% and 103%. Six biological replicates were considered for this analysis.

### Starch analysis

Starch content was only evaluated in the hypocotyls which are the main reserve organs. Starch was extracted and analyzed as previously reported^18^. Six biological replicates were considered for this analysis.

### Glucosinolate analysis

Extraction and analysis were performed as previously described Esparza *et al*.^14^ with some modifications. Briefly, extraction was performed by using freeze-dried samples in 70% methanol and supernatants were collected. Desulfoglucosinolates in each extract were collected by using anion exchange solid phase extraction columns (Agilent Bond Elut SAX). Eluates were analyzed in a 1290 Agilent UPLC (Santa Clara, CA,USA) equipped with a DAD UV-Vis detector and a Licrospher 100 RP-18 column (Merck, Darmstadt, 250 × 4.6 mm, 0.5 µm particle size) at 30 °C and 1 mL/min flow rate. The elution gradient used acetonitrile (A) and water (B) as follow: 2 to 5% A in 6 min, 5 to 7% A in 2 min, 7 to 21% A in 10 min, 21 to 29%A in 5 min and a final wash from 29 to 100% A in 2 min. Detection was performed at 229 nm. Six biological replicates were considered for this analysis.

### Phenolic compounds

Total soluble phenolic compounds were assayed with the method based on Folin-Ciocalteau’s phenolic reagent and spectrophotometrically determined as previously reported^19^, using 10 µL of glucosinolate extracts. Six biological replicates were considered for this analysis.

### Photosynthetic pigments

Pigments were extracted and analyzed as previously reported Huarancca Reyes *et al*.^20^. Six biological replicates were considered for this analysis.

### Leaf gas exchange and chlorophyll *a* fluorescence

Leaf gas exchanges and chlorophyll *a* fluorescence were simultaneously measured with a portable photosynthesis instrument (LI-6800, LI-COR Inc., NE, USA) fitted with the multiphase flash fluorometer (6800-01). All physiological measurements were performed on the central fully expanded leaflet of 4- and 7-month-old plants with three biological replicates. Conditions were set at CO_2_ concentration of 400 µmol mol^−1^, relative humidity ranging between 50 and 60%, leaf temperature of 25 °C and at light intensity of 1600 µmol m^−2^ s^−1^. Instantaneous measurements of CO_2_ assimilation rate, intercellular CO_2_ concentration, stomatal conductance and transpiration rate were determined as previously described^20^. The maximum PSII photochemical efficiency (*F*_*v*_/*F*_*m*_) was determined as *F*_*v*_/*F*_*m*_ = (*F*_*m*_ − *F*_0_)/*F*_*m*_, where *F*_*m*_ and *F*_0_ represent the maximum and the minimum fluorescence yield emitted by the leaves in the dark-adapted state, respectively^21^.

### Statistical analysis

Data were analyzed with STATISTICA for Windows v. 13.4.0 (Start-Soft, Inc., Tulsa, USA). Values presented are means ± standard error of at least three replicates. Following Bartlett’s test for homogeneity of variance, all data were subjected to two-way analysis of variance (ANOVA) with treatment (control and UV-block) and age of plants (4- and 7-month-old) as main factors. Significant differences among means were estimated at the level of *P* < 0.05 by using Tukey test. For all parameters, the assumption of normality distribution was confirmed by the Shapiro-Wilks test.

### Ethical statement

The study is in compliance with ethical standards.

## Results

### Biometric analysis

**Figure 1:**
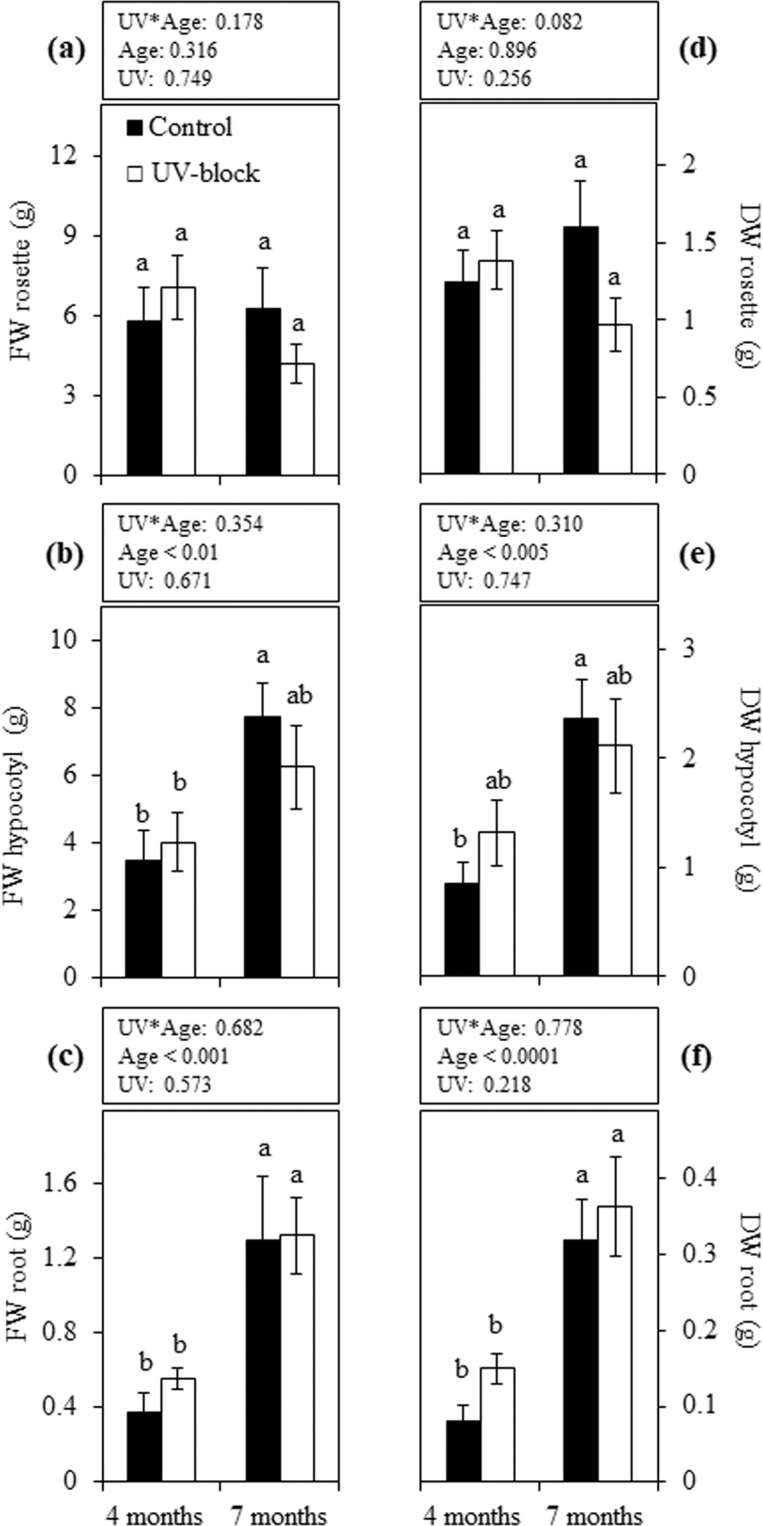
Biometric traits in maca plants grown under UV-transmitting and -blocking filters. Fresh (**a**–**c**) and dry weight (**d**–**f**) of 4- and 7-month-old plants grown under mountain ecosystem. Error bars represent the standard error of the mean (n = 6). The *P*-values indicate the significance of the effects of treatment (UV), plant age (Age) and their interaction (UV*Age), all calculated from a two-way ANOVA. Differences between means are indicated by different letters (*P* < 0.05). Control, UV-transmitting filter. UV-block, UV-blocking filter. FW, fresh weight. DW, dry weight.

**Figure 2:**
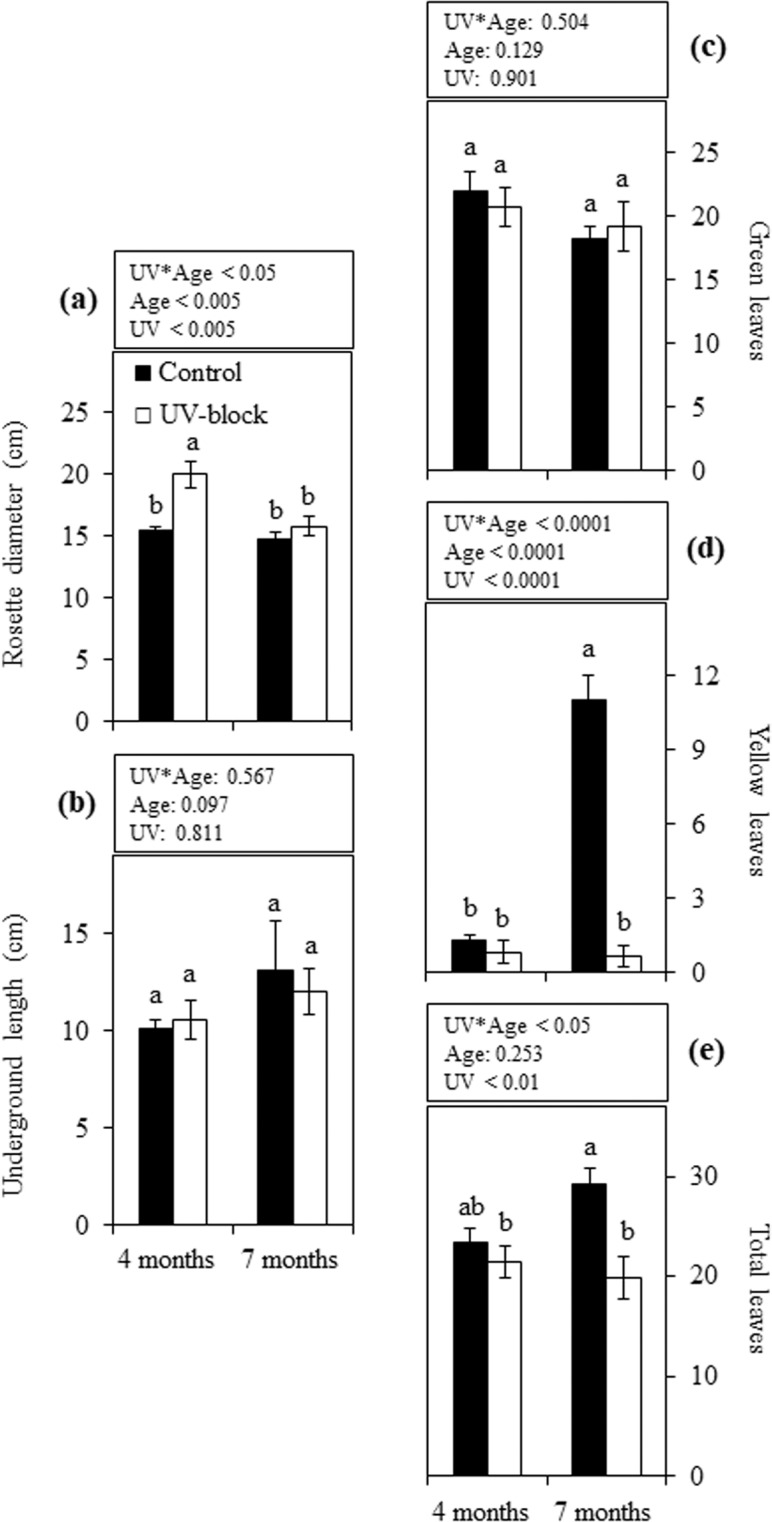
Additional vegetative parameters in maca plants grown under UV-transmitting and -blocking filters. Rosette diameter (**a**), length of underground organs (**b**), and green (**c**), yellow (**d**) and total (**e**) leaves number of 4- and 7-month-old plants grown under mountain ecosystem. Error bars represent the standard error of the mean (n = 6). The *P*-values indicate the significance of the effects of treatment (UV), plant age (Age) and their interaction (UV*Age), all calculated from a two-way ANOVA. Differences between means are indicated by different letters (*P* < 0.05). Control, UV-transmitting filter. UV-block, UV-blocking filter.

No differences were observed between UV-blocking and control conditions on rosette FW in maca plants at either 4 or 7 months of growth (Fig. 1a). Although the hypocotyl and root FW of these plants did not show a statistically significant difference between treatments, a significant increase in fresh biomass was observed at the latter stage with the exception of UV-blocked hypocotyls (Fig. 1b,c). Similar patterns were observed in the rosette, hypocotyl and root when comparing their respective DW (Fig. 1d–f). Significant differences between treatments were observed in the rosette diameter of 4-month-old plants, while no UV-treatment effect was observed when plants were 7 months old (Fig. 2a). The length of underground organs, as well as green leaf number, showed no significant differences between treatments or between plant ages (Fig. 2b,c). Yellow leaf number was unaffected by UV treatment in 4-month-old plants; however, at the latter stage control plants showed significantly higher yellow leaves than UV-blocked plants (Fig. 2d). Similarly, the total leaf count showed significant differences between treatments only when plants were 7 months old (Fig. 2e).

### Total soluble sugars

**Figure 3:**
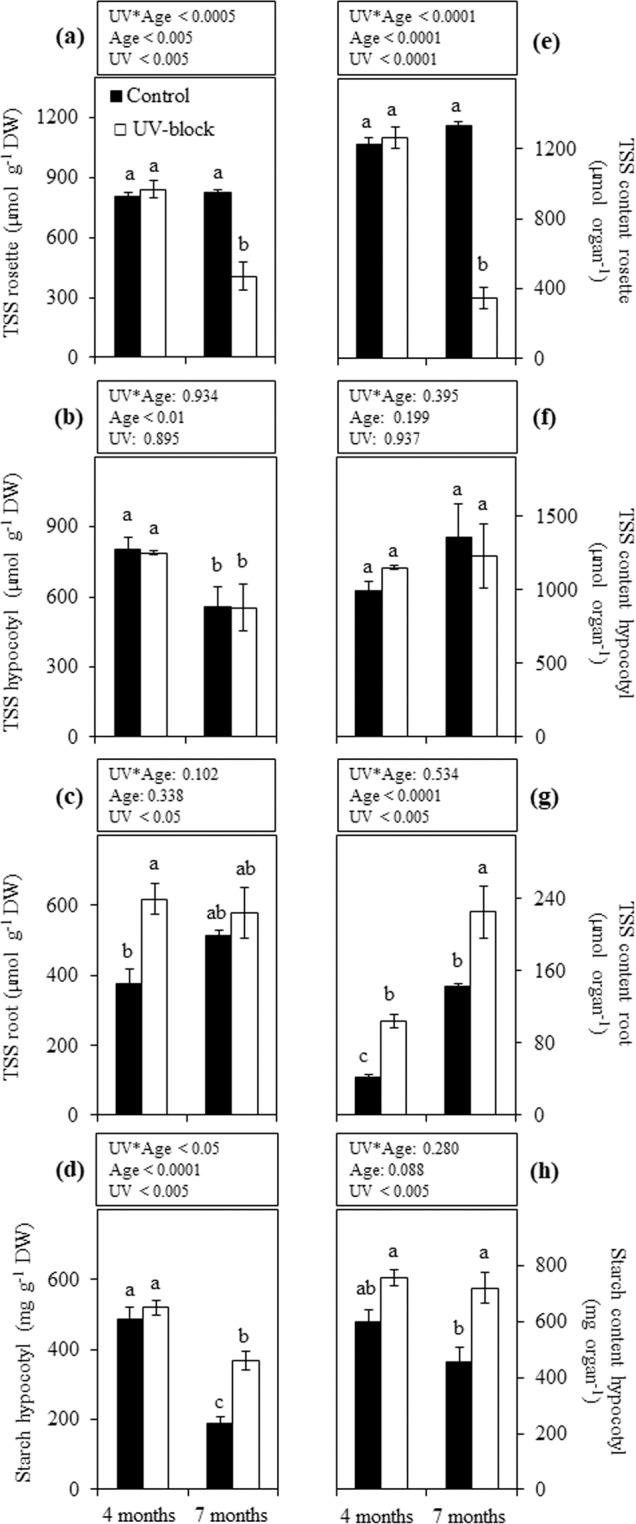
Carbohydrates in maca plants grown under UV-transmitting and -blocking filters. Concentration of total soluble sugars (TSS) expressed in μmol g^−1^ DW (**a**–**c**) and starch in mg g^−1^ DW (**d**) was determined in 4- and 7-month-old plants grown under mountain ecosystem. Total content of these carbohydrates was also calculated. (**e**–**h**) Error bars represent the standard error of the mean (n = 6). The *P*-values indicate the significance of the effects of treatment (UV), plant age (Age) and their interaction (UV*Age), all calculated from a two-way ANOVA. Differences between means are indicated by different letters (*P* < 0.05). Control, UV-transmitting filter. UV-block, UV-blocking filter. DW, dry weight.

TSS concentration in the rosettes of 4-month-old plants was similar between treatments; this level was maintained in 7-month-old control plants, while UV-blocked plants at the same developmental stage showed a significant decrease (Fig. 3a). A different pattern was observed in hypocotyls, which at 4-month-old contained higher TSS levels than 7-month-old plants without significant differences between treatments (Fig. 3b). Roots of 4-month-old plants showed significantly higher TSS when grown under UV-blocking than control condition, this difference was not observed when plants were 7 months old (Fig. 3c). In addition to concentration values, we also reported TSS as total content per organ due to significant observed differences in the biomass of organs between the treatments (Fig. 1). TSS content in rosettes (Fig. 3e) followed a similar pattern as when it was expressed as concentration per DW (Fig. 3a). Hypocotyls did not display significant differences between treatments and ages (Fig. 3f), while roots were significantly affected by UV-treatment and developmental stage (Fig. 3g).

### Starch content

The effect of UV-treatment on starch concentration was not observed in 4-month-old plants, while at 7 months the starch concentration in hypocotyls of plants grown under UV-blocking filters was significantly higher than in control plants (Fig. 3d). Report of starch as total content per organ showed similar UV-treatment effects as when it was expressed as concentration per DW, where significant differences were only observed in 7-month-old plants (Fig. 3h).

### Secondary metabolites

**Figure 4:**
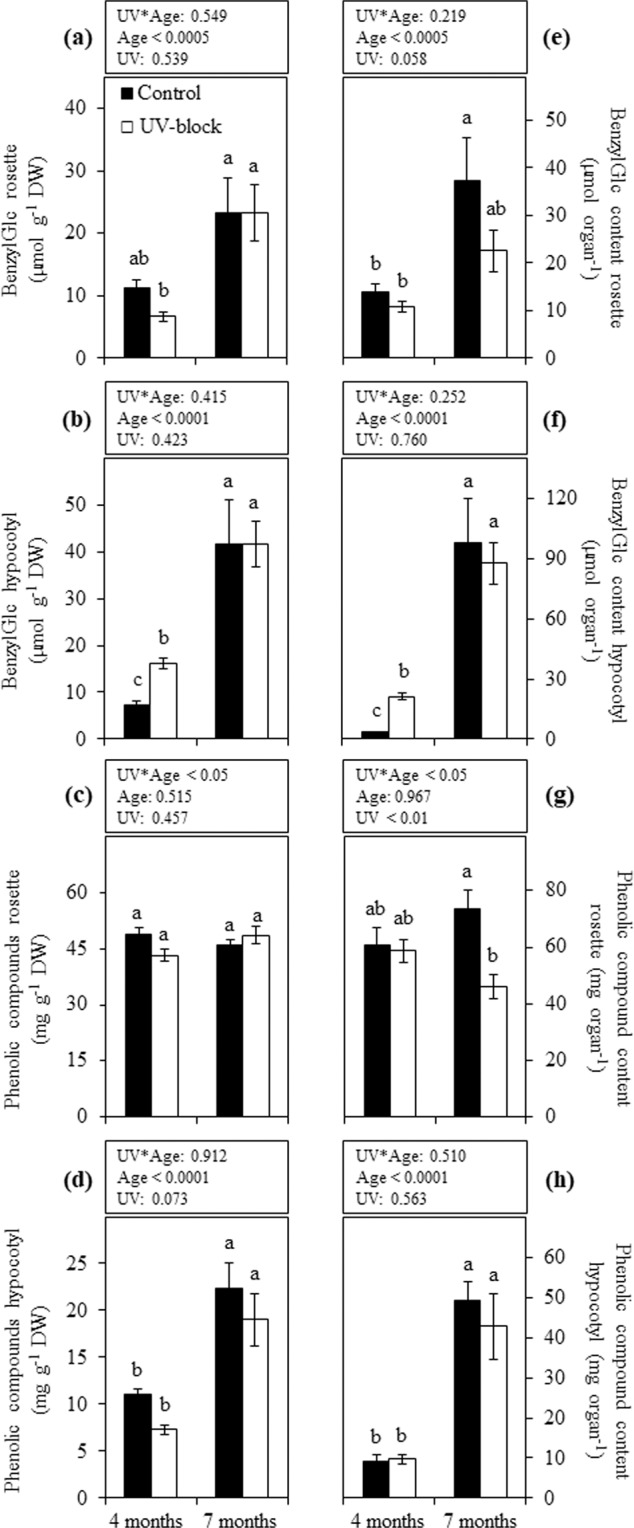
Secondary metabolites in maca plants grown under UV-transmitting and -blocking filters. Concentration of benzylglucosinolate expressed in μmol g^−1^ DW (**a**,**b**) and phenolic compounds in mg g^−1^ DW (**c**,**d**) was determined in 4- and 7-month-old plants grown under mountain ecosystem. Total content of these secondary metabolites was also calculated. (**e**–**h**) Error bars represent the standard error of the mean (n = 6). The *P*-values indicate the significance of the effects of solar UV (UV), plant age (Age) and their interaction (UV*Age), all calculated from a two-way ANOVA. Differences between means are indicated by different letters (*P* < 0.05). BenzylGlc, benzylglucosinolate. Control, UV-transmitting filter. UV-block, UV-blocking filter. DW, dry weight.

Benzylglucosinolate in rosettes of 4- or 7-month-old plants was not affected by the treatments, while the effect of plant age was more marked in UV-blocking than in control conditions with a significant increase at the latter stage of development (Fig. 4a). The concentration of benzylglucosinolate in hypocotyls of 4-month-old plants showed significant differences between treatments, in which those in control were significantly lower than in UV-blocking conditions. A subsequent significant increase in benzylglucosinolate was observed in 7-month-old plants, which did not show significant differences between treatments (Fig. 4b). Similar patterns were observed in rosettes and hypocotyls when benzylglucosinolate content was expressed as total µmol per organ (Fig. 4e,f). Total phenolic compounds in rosettes did not show differences in tissue concentrations between treatments and ages (Fig. 4c). In hypocotyls, significant differences were observed between stages of growth in plants under the same treatment, resulting in high levels of phenols in 7-month-old plants (Fig. 4d). A similar pattern was observed in the total phenol content of hypocotyls (Fig. 4h). The content of phenols in rosettes resulted in a clear effect between treatments only in plants at the latter stage of development, where those under UV-blocking filters had significantly lower phenol content than controls (Fig. 4g).

### Photosynthetic pigments

**Figure 5:**
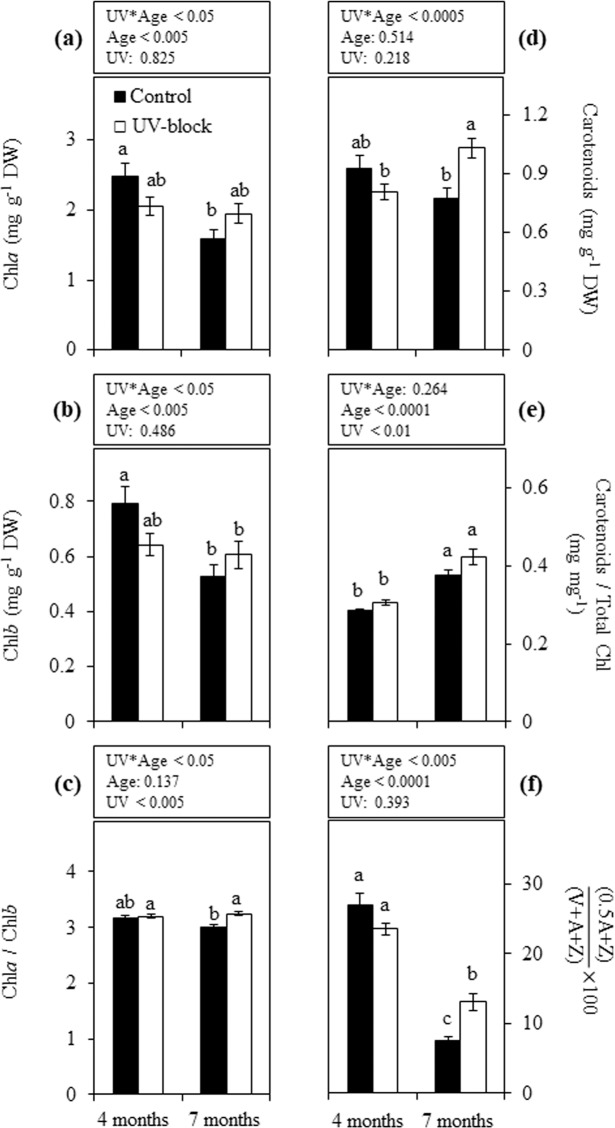
Photosynthetic pigments in maca plants grown under UV-transmitting and -blocking filters. Chl*a* (**a**), Chl*b* (**b**), Chl*a*/Chl*b* ratio (**c**), Total carotenoids (**d**), Total Carotenoids/Total Chl ratio (**e**) and de-epoxidation state (**f**) were determined in 4- and 7-month-old plants grown under mountain ecosystem. Error bars represent the standard error of the mean (n = 6). The *P*-values indicate the significance of the effects of treatment (UV), plant age (Age) and their interaction (UV*Age), all calculated from a two-way ANOVA. Differences between means are indicated by different letters (*P* < 0.05). Control, UV-transmitting filter. UV-block, UV-blocking filter. Chl, chlorophyll. A, antheraxanthin. Z, zeaxanthin. V, violaxanthin. DW, dry weight.

No effects were observed on Chl*a* concentration between treatments in 4- or 7-month-old plants. However, a significant decline was detected in plants under control conditions at 7 months, while this effect was not observed under UV-blocking conditions (Fig. 5a). Results for Chl*b* were similar to those of Chl*a* (Fig. 5b), with some differences in their ratio. Indeed, Chl*a*/Chl*b* ratios showed significant differences between treatments only in 7-month-old plants, where those grown under UV-blocking filters reported higher ratios than those under control condition (Fig. 5c). UV-treatment effects on total carotenoids were only observed at the latter stage, where plants grown under UV-blocking filters showed higher levels than those grown under control condition, and increase of carotenoids over time was only registered in plants under UV-blocking conditions (Fig. 5d). The ratio of carotenoids relative to total Chl did not show significant differences between treatments in plants at the same age, while the ratio in 7-month-old plants was highly increased with respect to 4 months under the same treatment (Fig. 5e). De-epoxidation state of xanthophyll cycle pigments in 4-month-old plants did not show differences between treatments, followed by a significant decrease when plants were 7 months old. At the latter stage, the de-epoxidation state in control plants was significantly lower than that of plants under UV-blocking condition (Fig. 5f).

### Leaf gas exchange and chlorophyll *a* fluorescence

**Figure 6:**
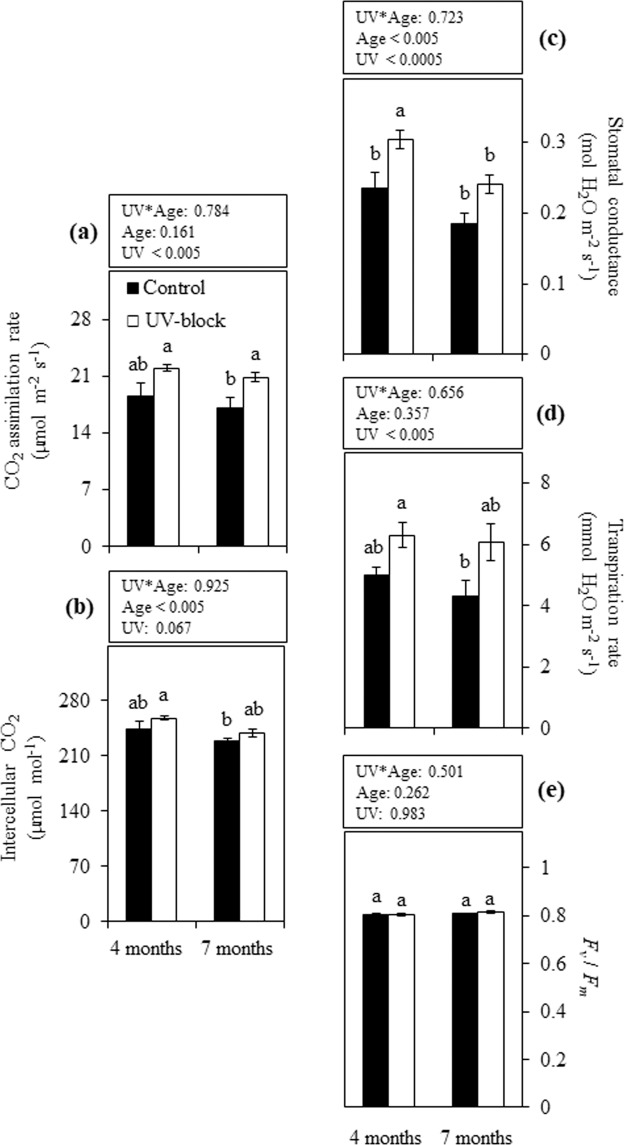
Leaf gas exchanges and chlorophyll *a* fluorescence in maca plants grown under UV-transmitting and -blocking filters. Parameters of CO_2_ assimilation rate (**a**), intercellular CO_2_ concentration (**b**), stomatal conductance (**c**), transpiration rate (**d**) and *F*_*v*_/*F*_*m*_ (**e**) were evaluated in 4- and 7-month-old plants grown under mountain ecosystem. Error bars represent the standard error of the mean (n = 3). The *P*-values indicate the significance of the effects of treatment (UV), plant age (Age) and their interaction (UV*Age), all calculated from a two-way ANOVA. Differences between means are indicated by different letters (*P* < 0.05). Control, UV-transmitting filter. UV-block, UV-blocking filter.

No effects were observed on CO_2_ assimilation rate between stages of growth under the same treatment. Although 4-month-old plants did not show statistically significant differences on CO_2_ assimilation rates between treatments, 7-month-old control plants assimilated significantly lower CO_2_ than plants under UV-blocking filters (Fig. 6a). Analysis of the intercellular CO_2_ concentration did not show significant differences between treatments and stages of growth (Fig. 6b). Similar patterns were observed on the transpiration rate (Fig. 6d). UV-treatment effects on stomatal conductance was only observed at 4 months, where plants under UV-blocking filters presented significantly higher levels than those under control condition, and decrease of stomatal conductance over time was only registered in plants under UV-blocking conditions (Fig. 6c). Values of *F*_*v*_/*F*_*m*_ in maca leaves did not show differences between treatments and ages (Fig. 6e).

## Discussion

In our previous study^15^ we found that maca grown in growth chamber showed differential UVB responses including a coordinated source-sink carbon allocation and a systemic phenotypical plasticity. In this study, we manipulated the solar UV radiation under field conditions in order to understand the influence of UV on physiological responses of maca plants over a complete growing season.

Outdoor experiments are much more complicated than indoor ones due to the involvement of different environmental factors, and thus, creating an intricate cross-talk between different signaling pathways in plants^22^. Here, sowing occurred at the beginning of summer with an UV index of 17, while samplings were performed at the start of autumn (4 months) with an UV index of 16 and at the start of winter (7 months, harvest) with an UV index of 11 (Supporting information, Fig. S2). According with our results, biomass of belowground organs did not show differences between treatments within the same sampling time, suggesting that biomass allocation to sink organs is not affected by UV. Previous studies using supplemental UVB in tuber crops showed that *Solanum tuberosum* decreases its tuber biomass after UVB exposure^23^, while the wild potato *Solanum kurtzianum* does not^24^, highlighting that the latter species is better adapted to high UVB levels. Thus, UVB effects on morphological changes are species-specific, and species originating from naturally high UV environments, such as maca, might tolerate better strong solar UVB irradiances^25^. Moreover, it is known that in herbaceous perennial or biennial plants, such as maca, store assimilates in the belowground organs before winter, which is a developmental program governed by various environmental cues^26^. Altogether, these observations suggest that the increase in belowground biomass in maca is predominantly linked to other environmental parameters rather than UV. However, looking at hypocotyl weights in more detail, there were significant UV-mediated differences in biomass accumulation rate found over the vegetative phase. Maca hypocotyls exhibited a significant increase in weight under control conditions, while this effect was not observed in plants grown under UV-blocking filters. These data suggest that UV radiation somehow modulates the investment of assimilates in storage tissue growth probably associated with UV-related response mechanisms, but not affecting the biomass allocation in hypocotyls during harvest.

In the case of aboveground organs, no differences were observed in their biomass either between treatments or over the vegetative growth phase. However, control plants showed a marked senescence acceleration and generation of new leaves, while plants grown under UV-blocking conditions maintained their green/yellow leaves. Interestingly, similar physiological responses were observed in maca plants subjected to UVB irradiation challenges in growth chamber^15^, suggesting that this coordinated remobilization and relocation of nutrients from old leaves to new leaves and storage organs is an UVB-related response in maca. Further studies must be performed in order to investigate the separate influences of PAR, UVB and UVA in the observed assimilates allocation between above and belowground organs in maca.

Plants are inevitably exposed to solar UV radiation, which may affect photosynthetic performance by alterations in photosystem II (PSII) photochemistry, pigment content (chlorophylls and carotenoids), RuBisCO activity and stomatal functions^27^. In this study, our results showed that UV radiation did not induce detrimental effects on the function of PSII as reflected in the consistent optimal values of *F*_*v*_*/F*_*m*_ in maca plants grown under control or UV-blocking condition and throughout their growing period. However, slight stress effects of solar UV were observed on CO_2_ exchange without association to possible alterations in the efficiency of PSII photochemistry as illustrated by the unchanged *F*_*v*_*/F*_*m*_ values. Our results showed no effects caused by UV and plant age in the intercellular CO_2_ concentration and transpiration rate, while a significant decrease in CO_2_ assimilation rate and stomatal conductance in control plants was detected at specific developmental stages (at 7- and 4-month, respectively). Stomata regulate the transpiration and CO_2_ uptake by leaves and its closure is related to the sense of internal/external environmental stimuli. For instance, cold temperatures and UVB radiation can induce the accumulation of abscisic acid (ABA) promoting stomatal closure through the activation of reactive oxygen species (ROS) and nitric oxide (NO)^28–30^. Maca plants experienced a temperate climate with strong UV levels up to the beginning of autumn, leading to a reduction of stomatal conductance in control plants probably due to a specific UVB signaling activation but without effects in CO_2_ assimilation rate. Differently, until harvest period, the minimum temperature decreases much more than UV levels, leading to a possible cold-induced stomatal closure in plants under UV-blocking conditions without effects on CO_2_ assimilation rate, while control plants maintained similar stomatal conductance as at early developmental stage. This suggests that UV may play an important role in cold acclimation, maintaining a consistent stomatal conductance and CO_2_ assimilation rate throughout the various seasons. Therefore, the strong UV radiation registered at high altitude did not affect the photosynthetic performance in maca plants, acting as a “good stress” rather than a “distress”^5^.

Changes in photosynthetic characteristics can be used as indicators to evaluate the sensitivity of plants in response to UV radiation^10,31^. Chlorophyll content in this work was not affected by plant age as illustrated in plants under UV-blocking conditions, while a significant decrease was registered in control plants. Intriguingly, UV did not affect the photosynthetic performance in maca plants, which lead us to speculate that chlorophylls reduction is not due to UV-induced degradation, but rather to UV-related morphological changes in leaves^8^ as reflected in the increment in leaf dry weight over the time.

Carotenoids are pigments that protect the photosynthetic apparatus from photooxidative damage due to several types of stress such as UVB^32^. Our results showed that carotenoids predominately change depending on the plant age rather than the UV radiation. In fact, control plants have more senescent leaves than plants grown under UV-blocking filters. Moreover, it is known that the de-epoxidation state (DEPS) of xanthophyll cycle pigments is related to the protection of PSII by quenching excited chlorophyll and protecting thylakoids from photodamage^33^. Here, DEPS at the beginning of autumn (4 months) was higher than at the beginning of winter (7 months) independent of the UV radiation, suggesting that de-epoxidized xanthophylls are mainly involved in the dissipation of excess of energy due to the strong photosynthetically active radiation (PAR) of sunlight at temperate temperatures. Autumn season was characterized by relative high solar radiation and low temperatures, which resulted in a strong reduction of DEPS probably due to the repressive effect that low temperature has on violaxanthin de-epoxidase activity^34^. Moreover, it was demonstrated that UV radiation negatively affects the biosynthesis and de-epoxidation of xanthophylls^35^. Accordingly, DEPS reduction due to low temperature was accentuated with UV, indicating that UV and low temperature may interact as accumulative stresses.

Regarding phenolic compounds, levels found in leaves were not affected by plant age, while only significant differences were observed at the beginning of winter in control plants which contain more phenolic compounds than those grown under UV-blocking conditions. Previous study^35^ revealed that UV radiation increases flavonoids (the largest group of phenolics compounds in plants) while inhibits xanthophyll biosynthesis and de-epoxidation, proposing that flavonoids complement xanthophylls in the chloroplasts protection to high doses of UV. Thus, these data suggest that maca plants under high solar UV might protect better their leaves by the investment of photosynthate in the synthesis of phenolic compounds rather than carotenoids, especially when leaf senescence takes place and new young leaves require more efficient protection. Differently, phenolic levels in hypocotyls were only affected by plant age rather than UV radiation, indicating that other environmental parameters regulate this accumulation as reported in some potato genotypes which present significant high phenols in their tubers in plants grown in low temperature regions^36^.

Glucosinolates are characteristic secondary metabolites of the Brassicaceae and are the main components in maca hypocotyls, of which aromatic glucosinolates such as benzylglucosinolate are found in high concentrations. Here, benzylglucosinolate in rosettes followed a similar pattern to that of phenolic compounds, but in lower concentration considering its molecular weight of 409 g mol^−1^. It was reported that CYP83A1 (a cytochrome P450 enzyme) is a key regulator in the crosstalk between phenolic and glucosinolate signaling pathways in Arabidopsis leaves^37^, suggesting a possible trade-off in the biosynthesis of these secondary metabolites in aboveground organs depending on their capacities to effectively counter the environmental stressors such as solar radiation and temperature due to seasonal variations. Benzylglucosinolate in hypocotyls increased over the experimental period, as it was previously demonstrated in maca hypocotyls of plants grown under mountain conditions during pre-harvest^38^. Interestingly, control plants showed a sharper increase in benzylglucosinolate than plants grown under UV-blocking conditions, suggesting that UV radiation is involved in the biosynthesis of this glucosinolate over the time in belowground organs. UVB and UVA radiation have been reported to enhance glucosinolates as well as phenolics by the induction of transcription factors involved in their biosynthetic pathways^39^. The mechanisms remain to be established, perhaps including signal transduction cascades by UVA- and UVB-specific photoreceptors and/or ROS-related signaling pathways.

Since the biosynthesis of secondary metabolites such as glucosinolates and phenolic compounds are strongly coupled with photosynthetic carbon fixation^40^, parameters such as the content and distribution of total soluble sugars (TSS) and starch in maca plants are relevant. In this study, starch degradation is induced in hypocotyls without a significant translocation of TSS from above to belowground organs over the time, as reflected in control plants. Interestingly, when UV radiation was blocked, there was a dramatic acceleration of TSS translocation to belowground organs reducing significantly the starch degradation. Since the photosynthetic performance was not affected by UV radiation, our hypothesis is that UV-related signaling may interfere with other environmental signaling such as the modulation of carbon allocation between source and sink organs due to low temperature. Accordingly, the ELONGATED HYPOCOTYL 5 (HY5) is a shoot-root mobile signal that regulates carbon fixation and translocation from shoot to root, and thus mediating homeostatic coordination of carbon and nitrogen metabolism at varying light fluence^41^. Moreover, HY5 is involved in multiple phytohormonal and environmental signaling inputs controlling plant growth and development. For instance, UVB photoreception generates UVR8 monomers that interact with COP1 stabilizing HY5 that in turn regulates UVB related target genes^6^. Similarly, it was reported that HY5 can be stabilized by the photoreceptor CRY that perceives UVA and blue light^42^. In this line and supporting our hypothesis, a recent genome study of maca identified several genes involved in low temperature and UVB response that were upregulated when plants grown at 4200 m of altitude^43^.

In conclusion, maca plants are notably adapted to high UV levels at high altitudes and respond to strong UV radiation by a coordinated remobilization and relocation of metabolites between source and sink organs via a possible UV signaling pathway. The UV-related signaling may interfere with and modulate response mechanisms to other environmental parameters due to seasonal variations allowing plants to acclimate under harsh environmental conditions. Thus, it will be interesting to elucidate the detailed mechanism of UV response in maca and how it modulates other environmental parameters-related pathways taking in consideration the great effort to sequence the genome of maca^44^.

## Supplementary information

Supplementary Information.
