# Supplementary material for: Physiological responses of maca (Lepidium meyenii Walp.) plants to UV radiation in its high-altitude mountain ecosystem
Source: Sci Rep. 2020 Feb 14;10:2654. doi: 10.1038/s41598-020-59638-4 (PMC7021813; doi:10.1038/s41598-020-59638-4)
Supplement: Supplementary file 1 — Supplementary Information. [file 41598_2020_59638_MOESM1_ESM.pdf]

Scientific Reports Supplementary Information

Article title: Physiological Responses of Maca (*Lepidium meyenii* Walp.) Plants to UV Radiation in its High-altitude Mountain Ecosystem

Authors: Huarancca ReyesT, Esparza E, Crestani G, Limonchi F, Cruz R, Salinas N, Scartazza A, Guglielminetti L, Cosio E

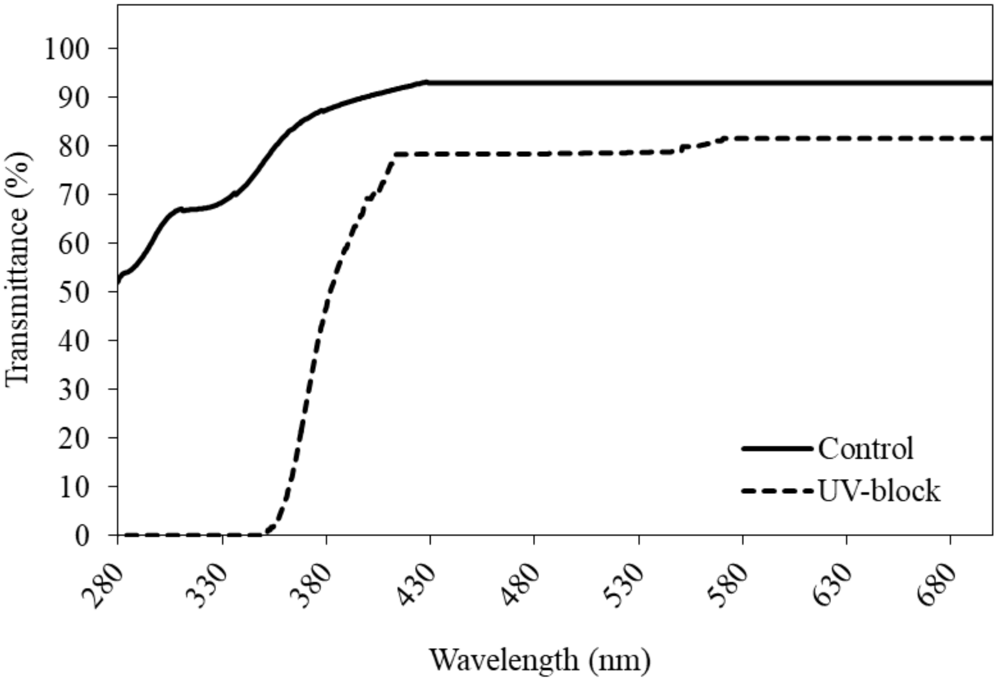

Supplementary Figure S1. Transmission spectra of UV-transmitting filter (Control, black line) and UV-blocking filter (UV-block, dashed line) used to cover the tunnels under field conditions.

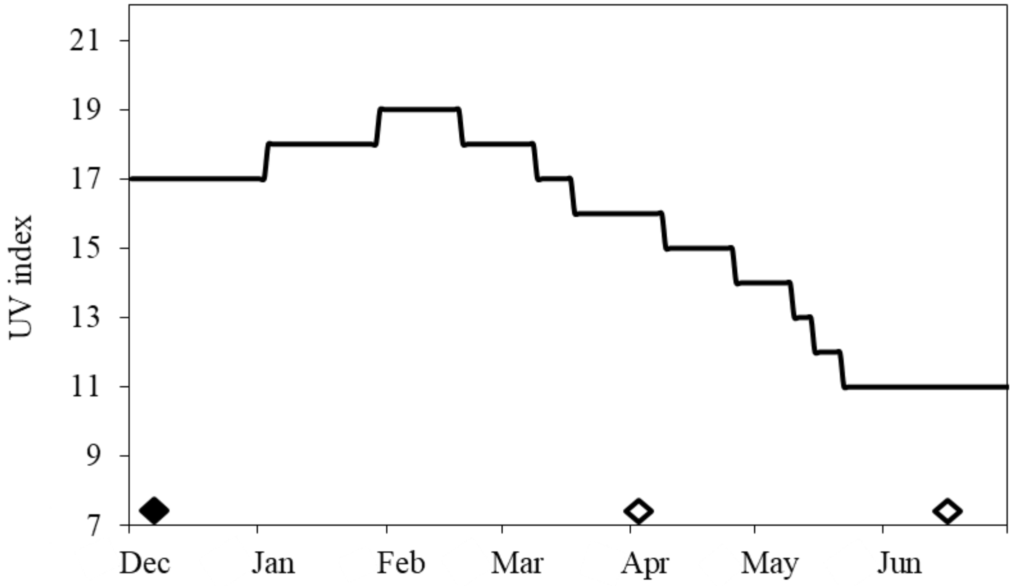

Supplementary Figure S2. Maximum daily erythemal UV index at the experimental site (Junín, Peru). Data were collected between December 2016 and June 2017. Sowing (closed diamonds) and sampling dates (open diamonds) are indicated at the bottom of the graph. For details, see Material and Methods.
